# Infective endocarditis and post-treatment bacteremia caused by oral microorganisms: a retrospective cohort study

**DOI:** 10.1007/s10096-026-05540-2

**Published:** 2026-05-16

**Authors:** Frederik Viktor Bang Jespersen, Simon Storgård Jensen, Merete Markvart, Henning Bundgaard, Peter L. Graversen, Emil L. Fosbøl

**Affiliations:** 1 Department of Odontology, University of Copenhagen, Copenhagen, Denmark; 2 Department of Oral & Maxillofacial Surgery, Copenhagen University Hospital, Copenhagen, Denmark; 3 Department of Cardiology, Copenhagen University Hospital, Copenhagen, Denmark

**Keywords:** Infective endocarditis, Index pathogen, Baseline characteristics, Oral microorganisms, Bacteremia, Relapse bacteremia

## Abstract

**Purpose:**

Infective endocarditis (IE) is a severe condition with risk of recurrence. Oral microorganisms (OM) are frequently implicated as index pathogens, yet patient characteristics, bacteremia with OM and relapse bacteremia after completion of IE treatment remain poorly described among these patients.

**Methods:**

This descriptive single-center retrospective cohort study included patients hospitalized with possible or definite IE at Copenhagen University Hospital. Patients were stratified by index pathogen into an OM and non-OM group. Baseline characteristics were described, and bacteremia with OM were assessed up to three years after completion of IE treatment using Aalen-Johansen cumulative incidence functions. Also, relapse bacteremia was evaluated in both groups.

**Results:**

Of 686 patients, 171 (25%) patients were categorized into the OM group, of which 83% had viridans group *streptococci* as index IE pathogen. Patients in the OM group had a median age of 62 years [IQR 49, 72] vs. 68 [IQR 59, 75] in the non-OM group. They had more often congenital heart disease, but had less often diabetes, chronic kidney disease and cardiac implantable electronic devices. The relapse bacteremia rate was 2%, occurring exclusively in the non-OM group. The three-year cumulative incidence of bacteremia with OM was 2.6%, 95% CI 0.1–5.1 and 2.8%, 95% CI 1.2–4.5, *p* = 0.88, in the OM and non-OM group, respectively.

**Conclusions:**

IE caused by OM occurred in younger and healthier patients. Relapse bacteremia was low and only observed in the non-OM group. The three-year cumulative incidence of bacteremia with OM occurred at comparable low rates across both groups.

**Supplementary Information:**

The online version contains supplementary material available at https://doi.org/10.1007/s10096-026-05540-2.

## Introduction

Despite advances in treatment, infective endocarditis (IE) remains associated with a substantial risk of recurrence [1]. Bacteremia after completion of IE treatment is a key marker for recurrence or reflects an ongoing infection predisposing the patient to a new IE event [2]. The causes of bacteremia are diverse, including urinary tract infections, catheter-related infections and recurrent IE [1–3]. However, the potential of the oral cavity to be a source of bacteremia with oral microorganisms (OM) has also been recognized for more than a century [4], as approximately 20% of all IE cases are caused by viridans group *streptococci*, a well-known oral bacterial group [5]. Poor oral hygiene and gingival inflammation are risk factors for transient bacteremia with OM [6], and maintaining sufficient oral hygiene may be the most important modifiable factors for preventing IE in patients with a history of IE [6].

Viridans group *streptococci* are traditionally regarded as the main pathogens in IE of oral origin [4], but the oral cavity contains a complex and diverse microbiota, with numerous species capable of causing IE [7]. Knowledge about patient characteristics in patients with OM as index IE pathogen remains limited. Existing research has primarily focused on the broad category of *Streptococci*, or viridans group *streptococci*, leaving other OM with limited attention [1, 8]. Furthermore, whether patients with OM index IE pathogens experience more frequent episodes of bacteremia with OM or relapse bacteremia after completion of IE treatment is poorly described.

Therefore, this descriptive retrospective cohort study aimed to describe baseline patient characteristics and assess the occurrence of all-cause bacteremia, bacteremia with OM and relapse bacteremia after completion of IE treatment, stratified by OM and non-OM index IE pathogens. Addressing this may help identifying patient characteristics relevant for follow-up and preventive strategies, as a better understanding of recurrent episodes of bacteremia and characteristics of patients is important for improving care after hospitalization.

## Methods

### Study design and setting

This descriptive single-center retrospective cohort study examined patients hospitalized with IE at Copenhagen University Hospital, Denmark from January 1, 2016 to December 31, 2021. Patients were stratified according to the index IE pathogen into an OM group and a non-OM group. Baseline characteristics were collected during hospitalization, and all patients were followed for a three-year period after completion of IE treatment for the occurrence of bacteremia. Bacteremia was categorized as all-cause bacteremia, bacteremia with OM and relapse bacteremia. Bacteremia analyses between groups were exploratory and unadjusted for confounding. Permission to perform the study and to handle the data was granted by the Danish Data Protection Agency (P-2020-92). The study followed the Strengthening the Reporting of Observational Studies in Epidemiology reporting (STROBE) guidelines [9].

### Data source and participants

Patients hospitalized at Copenhagen University Hospital were identified through the National Danish Endocarditis Studies (NIDUS) registry. The NIDUS registry contains individual patient data of all patients with definite or possible IE between January 1, 2016, and December 31, 2021 and has previously been described in detail [10]. All patients in the NIDUS registry have had possible or definite IE according to the modified Duke criteria and the European Society of Cardiology 2015 modified diagnostic criteria [11, 12]. All data have been registered retrospectively in a standardized web-based case report form using Research Electronic Data Capture (REDCap).

### Variables

Data extracted from the NIDUS registry included baseline information from hospitalization such as patient demographics, medical history, comorbidities, microbiological findings including the most likely primary IE index pathogen and culture or PCR analysis from extracted valve tissue, predisposing risk factors, complications, valve involvement, functional status and surgical interventions. Data regarding bacteremia were collected from the electronic medical record system.

### Patient categorization according to microbiological etiology

Patients were categorized based on the most likely primary IE index pathogen variable from the NIDUS registry, determined through blood culture findings. If this variable was missing, the microbiological etiology was classified according to culture or PCR analysis from extracted valve tissue. Patients were then categorized into the following groups: *Staphylococcus aureus*, OM, Other *Streptococcus spp.*,* Enterococcus spp.*, Coagulase-negative *Staphylococci (*CoNS*)*, Other microorganisms, and Unknown pathogens.

The OM group refers to species associated with the oral cavity that are common in dental plaque, or gingival and periodontal pockets, which were registered in the Human Oral Microbiome Database [13]. Typical species in the OM group include viridans group *streptococci*,* Aggregatibacter spp.*,* Abiotrophia spp.*, and *Gemella spp.* all of which are all listed in Table 1.

The groups of *Staphylococcus aureus*, Other *Streptococcus spp.*,* Enterococcus spp.*, CoNS and Other microorganisms were pooled as a non-OM group and compared to the OM group. Patients in the unknown pathogen group were excluded for further analysis.

### Outcomes and exposure

The primary outcome was first-time bacteremia with OM within three years after completion of IE treatment. Secondary outcomes were all-cause bacteremia and relapse bacteremia after completion of IE treatment. Also, baseline patient characteristics were described. All patients were stratified by IE index pathogen.

### Bacteremia analyses and definitions

Bacteremia events were identified through manual review of the Danish Microbiology Database (MiBa), which contains all microbiology results for Danish citizens [14]. An event was defined as ≥ 2 positive peripheral blood cultures for the same organism obtained within 24 h, to reduce the risk of misclassification due to contamination. Multiple episodes were recorded if a new event occurred after discharge from the previous event. Events were registered only if patients had contact with the Danish healthcare system after completion of IE treatment and had a blood culture sample obtained.

The first episode of bacteremia was considered the event of interest for cumulative incidence functions, and follow-up was up to three years after completion of IE treatment, ending no later than December 31, 2024. A three-year follow-up period was chosen to include late onset bacteremia, ensure enough follow-up time and align with previous IE studies. Patients who died within one week after completion of IE treatment or had lifelong antibiotics planned at discharge were excluded from bacteremia analyses.

Bacteremia was categorized as all-cause bacteremia, bacteremia with OM and relapse bacteremia. All-cause bacteremia was defined as the occurrence of bacteremia caused by any pathogen. Bacteremia with OM was defined as bacteremia caused by any microorganism listed in the Human Oral Microbiome Database [13] as primarily residing the oral cavity. Relapse bacteremia was defined as bacteremia occurring within six months after completion of IE treatment caused by the same pathogen as the index IE pathogen. Bacteremia with OM and relapse bacteremia could overlap, and a bacteremia episode did not in itself imply recurrence of IE.

### Statistical analyses

The statistical analyses were performed using RStudio Version 2024.12.1 + 563. Categorical variables were summarized as frequencies and percentages. The comparison of baseline characteristics between groups included using Chi-square or Fisher’s exact tests for categorial data. Continuous data were presented as medians with interquartile ranges (IQR) for non-normally distributed data and assessed using Mann–Whitney U test. Multiple comparison correction was applied for comparison of baseline characteristics using the Benjamini–Hochberg method.

For bacteremia analyses, cumulative incidence functions for the first episode of bacteremia with OM and all-cause bacteremia were estimated using the Aalen-Johansen method. All-cause death without prior bacteremia was treated as a competing risk during the three-year follow-up period, and patients alive and free of bacteremia were censored at three years after completion of IE treatment, or on the last date available for follow-up on 31 December 2024. The unadjusted cumulative incidence functions were compared between groups using Gray’s test.

## Results

### Study population

**Fig. 1:**
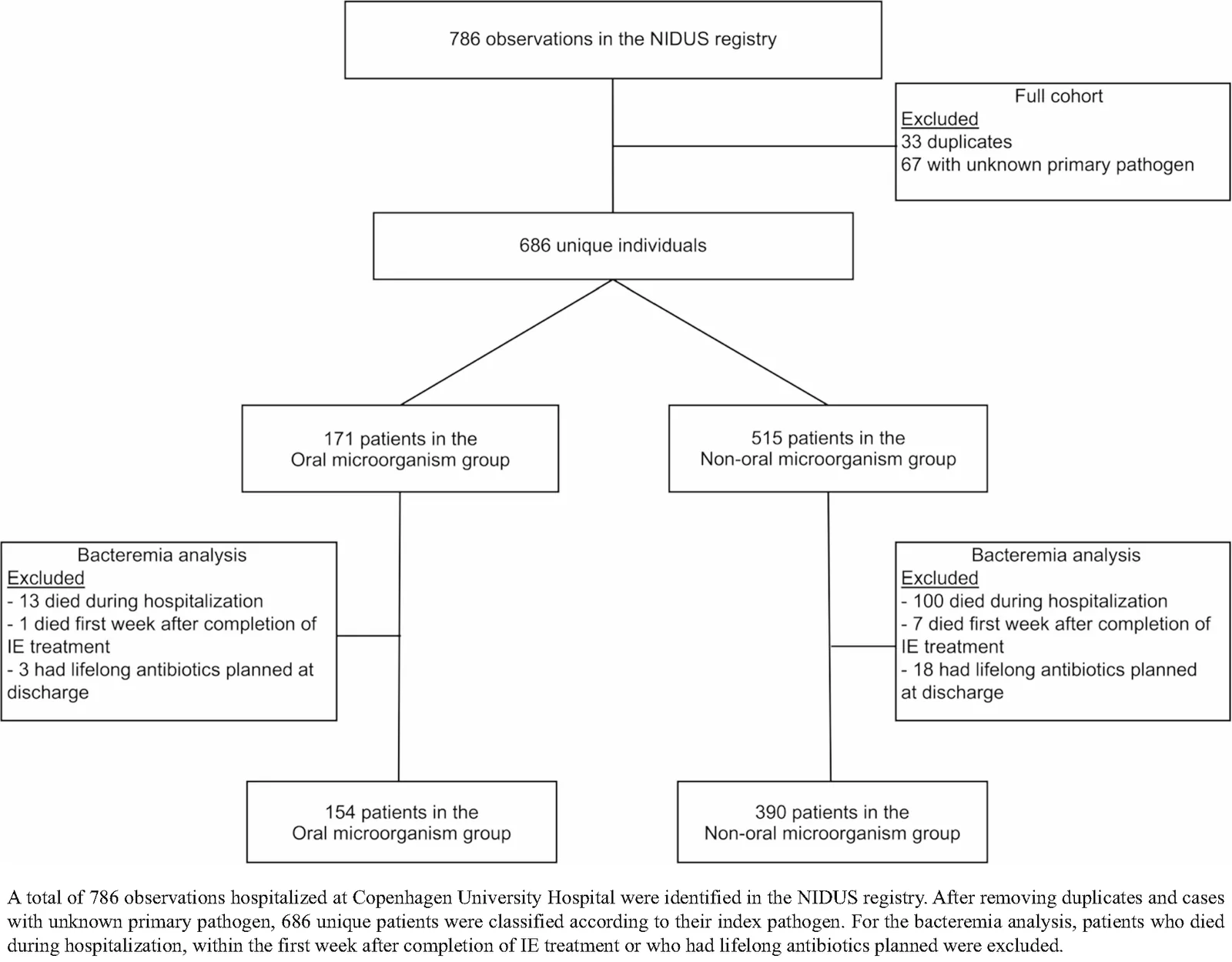
Flow of patients

A total of 686 patients were included (Fig. 1). The unknown pathogen group initially comprised 88 blood-culture negative patients. Of these, 30 were reclassified based on bacterial growth and three based on PCR analysis of extracted tissue or prosthetic material. However, 11 patients had a primary pathogen recorded as ‘Another’ and one as ‘HACEK’ (Supplementary S1), all unidentifiable at the genus level, resulting in 67 patients excluded for further analysis (Fig. 1).

### Categorization by index pathogen

The patients were categorized into the following groups: *Staphylococcus aureus* (*n* = 218, 32%), OM (*n* = 171, 25%), *Enterococcus* spp. (*n* = 98, 14%), other *Streptococcus* spp. (*n* = 90, 13%), CoNS (*n* = 73, 11%) and other microorganisms (*n* = 36, 5%), detailed in Supplementary S1. Multiple pathogens were registered in one patient from the CoNS group who had *Staphylococcus epidermidis*,* Staphylococcus petrasii* and *Staphylococcus hominis* registered. Within the OM group, 142 patients (83%) had viridans group *streptococci* as index pathogen, detailed in Table 1.

**Table 1:** Distribution on infective endocarditis index pathogens in the oral microorganism group

Species in the OM group	Number reported
*Streptococcus mitis*	60
*Streptococcus sanguinis*	40
*Streptococcus anginosus*	14
*Streptococcus mutans*	10
*Streptococcus gordonii*	6
*Streptococcus salivarius*	6
*Aggregatibacter aphrophilus*	5
*Haemophilus parainfluenzae*	5
*Streptococcus oralis*	4
*Abiotrophia defectiva*	4
*Granulicatella adiacens*	3
*Cardiobacterium hominis*	3
*Aggregatibacter actinomycetemcomitans*	2
*Rothia dentocariosa*	2
*Gemella haemolysans*	2
*Granulicatella elegans*	1
*Streptococcus constellatus*	1
*Streptococcus cristatus*	1
*Gemella morbillorum*	1
*Gemella bergeri*	1

Abbreviations: *OM*, Oral microorganism

### Baseline characteristics

Patients in the OM group were younger, with a median age of 62 years [IQR 49, 72] vs. 68 [IQR 59, 75] in the non-OM group (Table 2). The OM group patients had a lower proportion of diabetes, chronic kidney disease, immunosuppressant drug use and cardiac implantable electronic devices (Table 2). They required less assistance during hospitalization, but had more often congenital heart disease, pulmonary valve involvement and dental procedure performed within three months before hospitalization. Also, cardiac surgery and an oral examination during hospitalization were more often conducted in the OM-group (Table 2).

**Fig. 2:**
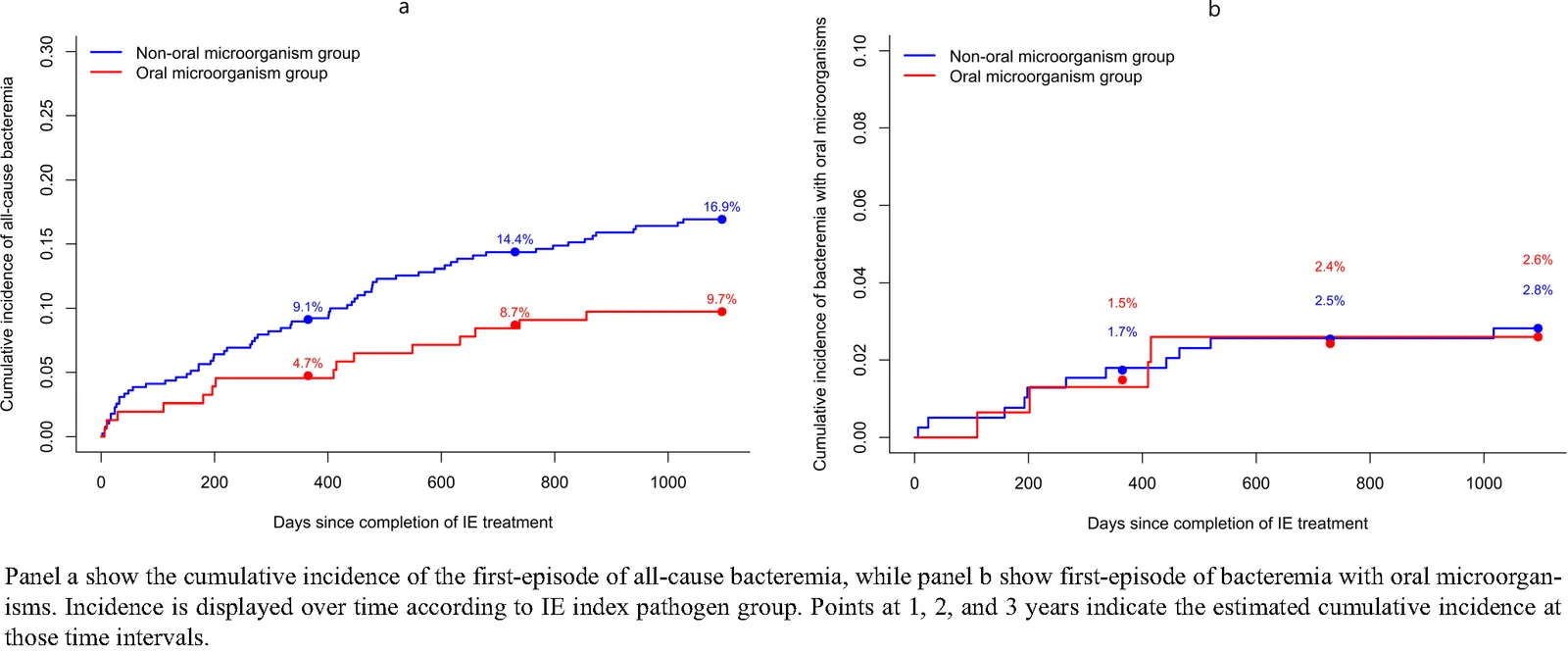
Cumulative incidence function of all-cause bacteremia and bacteremia with oral microorganisms stratified by index pathogen

### Bacteremia analyses

A total of 544 patients were eligible for bacteremia analyses (Fig. 1). The baseline characteristics and distribution of microorganisms were comparable to the full cohort (Supplementary S2 and Supplementary S3). The OM group more often had pulmonary valve involvement, and more frequently had a dental focus identified and less frequently had a focus in the urinary tract or skin (Supplementary S2). Information on how the focus of infection was managed during hospitalization was not registered.

All patients had the possibility of up to three years of follow-up, except for 13 patients who completed IE treatment after 31 December 2021. If these patients did not experience death or bacteremia, they were censored at their last available follow-up on 31 December 2024. This resulted in a minimum possible follow-up time of two years and nine months.

During the follow-up, the 544 patients contributed 1424 person-years of which 995 person-years were from the non-OM group and 430 person-years from the OM group. A total of 81 patients (15%) experienced all-cause bacteremia after completion of IE treatment. Multiple episodes of all-cause bacteremia were recorded in 33 patients (6%) resulting in a total of 145 episodes. The incidence rate of all-cause bacteremia per 100 person-years was 11.6 (CI 9.6–13.9) in the non-OM group and 7 (CI 4.9–10) in the OM group (Table 3).

### First episode of all-cause bacteremia

The median time to the first episode of all-cause bacteremia was longer in the OM group than in the non-oral group (Table 3), and the three-year cumulative incidence of all-cause bacteremia was 16.9% (CI 13.2–20.7) in the non-OM group and 9.7% (CI 5-14.4) in the oral group (Fig. 2 and Table 3). Unadjusted comparisons showed higher cumulative incidence of all-cause bacteremia in the non-OM group (*p* = 0.03).

### Relapse bacteremia

In 25 patients (5%), the first bacteremia episode was caused by the same pathogen as the index IE pathogen (Supplementary S4). This group consisted of two patients from the OM group and 23 patients from the non-OM group. Among these, bacteremia occurred within 6 months after completion of IE treatment in 11 patients, all from the non-OM group. Consequently, no relapse bacteremia was observed among patients in the OM group.

### First episode of bacteremia with OM

Bacteremia with OM occurred in 15 patients (3%). The three-year cumulative incidence of bacteremia with OM was 2.6% (CI 0.1–5.1) in the OM-group and 2.8% (CI 1.2–4.5) in the non-OM group (Fig. 2 and Table 3), and the dominant microorganisms were *Streptococcus mitis* and *Streptococcus sanguinis* (Supplementary S4). Unadjusted comparison showed no difference in cumulative incidence of bacteremia with OM between groups (*p* = 0.88).

## Discussion

We examined patients with IE and their characteristics by OM and non-OM index IE pathogen. This study had several major findings. First, the baseline characteristics in the OM group revealed patients that represented 25% of the cohort, were younger, had fewer comorbidities, and had a better functional status, but more frequently had congenital heart disease. Second, the rate of relapse bacteremia was low and exclusively happened in the non-OM group. Third, bacteremia with OM seemed to occur at similar rates in both groups over a three-year period.

Few studies have examined the baseline characteristics and bacteremia with detailed consideration to microorganisms that primarily reside in the oral cavity as index IE pathogen. Previous research has typically categorized microorganisms into broader groups such as *Streptococci* or viridans group *streptococci* [1, 15–18]. Using the Human Oral Microbiome Database in this study to classify OM, a more precise categorization than traditional streptococcal groupings was performed. While these microorganisms do not exclusively reside the oral cavity, they are particularly common in dental plaque, or gingival and periodontal pockets. Although viridans group *streptococci* were dominant in the OM group, this classification approach captured additional OM such as *Aggregatibacter*, *Gemella*, and *Abiotrophia*, which highlights the heterogeneity of OM implicated in IE. However, the classification of microorganisms as OM and non-OM is still not uniform, as multiple species like *Enterococcus faecalis*, which is generally considered a non-OM, also can be isolated from the oral cavity and associated with oral disease [19].

The findings in this study confirm that patients with OM as IE index pathogen differ from patients with non-OM. The species of *Staphylococcus aureus*, Beta-hemolytic *streptococci* and *Enterococcus* have previously been associated with a higher age and higher comorbidity burden [17, 20, 21], and the younger, healthier patients in the OM group is therefore not surprising. The higher prevalence of congenital heart disease has also been reported previously among IE patients caused by viridans group *streptococci* [22], and it is a known risk factor for pulmonary valve involvement which could explain the higher involvement rate of this in the OM group [23]. Speculatively, these findings could indicate that IE caused by OM may often result from structural cardiac anomalies rather than systemic vulnerability. This distinction could be clinically relevant, because it indicates two different ways in which IE can develop. One which is driven by anatomical predisposition and bacteremia with OM, and one by systemic vulnerability.

All-cause bacteremia occurred in 15% of the entire cohort within the three-year follow-up period, and both the incidence rate and the unadjusted three-year cumulative incidence were higher in the non-OM group. The cumulative incidence curves differed significantly between the two groups over the three-year follow-up. However this estimate is unadjusted, and the observed difference is likely caused by confounding including higher age, greater comorbidity burden and cardiac device prevalence in the non-OM group, which all predispose to recurrent infections [3].

In 2% of all patients, relapse bacteremia was observed and occurred exclusively in the non-OM group. One possible explanation for this is that IE caused by OM may often result from transient bacteremia [6], rather than a persistent infectious focus. However, several factors such as the anatomical localization of an embolic event and the focus of infection may influence relapse risk. We found no differences in embolism localization were between groups, but the OM group were more frequently associated with suspected dental focus and less often with urinary tract or skin foci. However, these findings should be interpreted with caution, as the suspected focus of infection was based on clinical assessment, and microbiological data. This estimation on the presumed focus has the consequence that the identified microorganism itself could influence the attribution of focus of infection. Information on how the focus of infection was managed during hospitalization was not registered, and the potential influence on relapse bacteremia could not be evaluated.

Interestingly, episodes of bacteremia with OM occurred at comparable low rates in both groups. This indicates that bacteremia with OM is clinically relevant among IE patients regardless of index IE pathogen. Given that increased frequency of transient bacteremia with OM is well documented in patients with gingival inflammation [6], and given that gingival inflammation is among the most prevalent condition worldwide [24], this finding suggests that exposure to OM bacteremia represents a continuous background risk after IE treatment. Currently, the guidelines of the American Heart Association and European Society of Cardiology highlight the importance of oral health in IE prevention [2, 25], and our findings therefore support this recommendation across IE patients regardless of index pathogen, where integration of oral assessment after discharge and long-term care could be considered. Gingival inflammation is a preventable and reversible condition that can be effectively managed through oral hygiene measures. Therefore, an oral assessment should include preventive oral care and hygiene instructions, and all IE patients regardless of index pathogen should be encouraged to seek regular professional dental follow-up in accordance with the recommendations of the European Society of Cardiology [2, 25].

In Denmark, current in-hospital oral assessment of IE patients typically consists of a single clinical and radiographic oral examination for all IE patients, especially for those with relevant index pathogens or scheduled for cardiac surgery [26]. This corresponds with our observation of a higher frequency of cardiac surgery and in-hospital oral examinations among patients with OM. There are no national guidelines on dental treatment of IE patients, except for elective patients undergoing cardiac surgery [27]. However, these guidelines primarily focus on removing oral infectious foci and do not emphasize preventive oral care or hygiene instructions. The impact of a single in-hospital oral examination and dental treatment remains unclear, especially whether oral findings influence decisions such as dental extractions and how these relate to the index pathogen category. Given that current practice prioritizes surgical removal of oral infectious foci, future studies should explore the role of an in-hospital oral examination and preventive strategies in reducing bacteremia with OM.

## Strengths and limitations

This study has several strengths. The NIDUS registry is a registry with clinical information and detailed individual data on IE patients, where a high percentage of agreement in critical variables among data collectors have been found [28]. Furthermore, no patients were lost to follow-up, as the unique identification number used in Denmark ensured accurate registration of mortality. Also, the use of the nationwide MiBa database minimized outcome misclassification.

However, this study also has several limitations related to its single-center descriptive and retrospective design. First, the single center design at a tertiary referral hospital introduces selection bias, as the group of patients is likely to represent a more complex group of patients compared to IE patients in general. This may limit the generalizability of the findings to other settings. Also, selection bias may have occurred, as only patients with a recorded ICD-10 diagnosis of IE were included, and patients who died before diagnosis were missed. The retrospective design introduces the risk of missing and incomplete data, as the study relied on data obtained from medical records. As an observational study without hypothesis testing or adjustment for confounders, causal relationships cannot be established. Furthermore, important factors such as oral health status at hospitalization and events during follow-up such as hospitalization, diseases, dental treatment and post-discharge antibiotic use were not available and may have influenced the occurrence of bacteremia. The absence of bacterial sequencing when analyzing blood samples may have resulted in missing causative pathogens.

## Conclusion

Patients with OM as index pathogen represented 25% of all IE cases and were observed in younger, healthier patients with better functional status, whereas non-OM were associated with older age and a greater comorbidity burden. There was a low rate of relapse bacteremia with no episodes among patients in the OM group, and bacteremia with OM occurred at comparable low rates in both groups, although the low number of events warrants cautious interpretation.

**Table 2:** Baseline characteristics stratified by index pathogen

Characteristics	Total	OM-group	Non-OM group	Significant *p*-values
Patients, n	686 (100%)	171 (25%)	515 (75%)	
Men	514 (75%)	132 (77%)	384 (75%)	
Age, years, IQR	67 [56, 75]	62 [49, 72]	68 [59, 75]	< 0.001*
Weight, kilogram, IQR^†^	86 [70, 111]	82 [67, 109]	88 [72, 111]	
Comorbidities				
Chronic kidney disease	129 (19%)	12 (7%)	117 (23%)	<0.001*
Dialysis†	56 (8%)	4 (2%)	52 (10%)	
Diabetes	157 (23%)	19 (11%)	138 (27%)	< 0.001*
COPD	95 (14%)	14 (8%)	81 (16%)	
Prior malignant disease	76 (11%)	14 (8%)	62 (12%)	
Active malignant disease	46 (7%)	7 (4%)	39 (8%)	
HIV/AIDS	7 (1%)	2 (1%)	5 (1%)	
Liver disease	31 (5%)	9 (5%)	22 (4%)	
Immunosuppressant drugs	76 (11%)	11 (6%)	65 (13%)	0.03*
Known heart failure	135 (20%)	22 (13%)	113 (22%)	
Native valve disease	72 (11%)	27 (16%)	45 (9%)	
Congenital heart disease	75 (11%)	39 (23%)	36 (7%)	<0.001*
Previous CIED	139 (20%)	20 (12%)	119 (23%)	0.005*
Previous heart valve surgery	217 (32%)	55 (32%)	162 (31%)	
Prior IE episode	44 (6%)	6 (4%)	38 (7%)	
Hospital stay, days, IQR	43 [30, 52]	37 [27, 47]	44 [32, 54]	< 0.001*
IE characteristics				
Localization				
Aortic valve	377 (55%)	97 (57%)	280 (54%)	
Mitral valve	281 (41%)	72 (42%)	209 (41%)	
Tricuspid valve	32 (5%)	5 (3%)	27 (5%)	
Pulmonary valve	33 (5%)	17 (10%)	16 (3%)	<0.001*
Pacemaker cord†	99 (14%)	12 (7%)	87 (17%)	
Myocardium†	15 (2%)	5 (3%)	10 (2%)	
Prosthetic valve	186 (27%)	49 (29%)	137 (27%)	
Modified DUKE criteria				
Definite IE	646 (94%)	150 (88%)	496 (96%)	<0.001*
Possible IE	40 (6%)	21 (12%)	19 (4%)	<0.001*
Complications during hospitalization				
Embolism	117 (17%)	22 (13%)	95 (18%)	
Heart failure	54 (8%)	18 (11%)	36 (7%)	
Conduction abnormalities	99 (14%)	31 (18%)	68 (13%)	
Persistence of bacteremia	49 (7%)	7 (4%)	42 (8%)	
Worsening valvular	113 (17%)	38 (22%)	75 (15%)	
insufficiency	107 (16%)	17 (10%)	90 (17%)	
Sepsis	79 (12%)	12 (7%)	67 (13%)	
Dialysis	197 (29%)	47 (27%)	150 (29%)	
Admission to ICU	402 (59%)	114 (67%)	288 (56%)	0.03*
Heart-surgery				
Functional status during hospitalization Walking aid necessary Patient-lift necessary Enteral/parenteral nutrition	379 (55%) 201 (29%) 216 (32%)	69 (40%) 31 (18%) 38 (22%)	310 (60%) 170 (33%) 178 (35%)	< 0.001* 0.002* 0.02*
Behavioral factors Active smoking Active drinking Active IV abuse	149 (22%) 457 (67%) 16 (2%)	33 (19%) 116 (68%) 4 (2%)	116 (23%) 341 (66%) 12 (2%)	
Dental procedure within three months before admission^†^	57 (9%)	25 (15%)	32 (6%)	0.002*
In hospital oral examination	396 (58%)	124 (73%)	272 (53%)	< 0.001*
In hospital death	113 (16%)	13 (8%)	100 (19%)	0.001*

Abbreviations: *OM*, Oral microorganism; *IE*, Infective endocarditis; *COPD*, Chronic obstructive pulmonary disease; *CIED*, Cardiac Implantable Electronic Device; *ICU*, Intensive Care Unit; *IV*, Intravenous

^*^Test results of Chi-square, Fisher’s exact, or Mann-Whitney U tests when appropriate. Significance is reported at *p* < 0.05 with Benjamini–Hochberg adjusted *p*-values

^†^Variable with ≥ 2% missing value

**Table 3:** Bacteremia analysis stratified by index pathogen

Bacteremia analysis, Outcome	OM group	Non-OM group
Patients, n	154 (28%)	390 (72%)
Overall bacteremia episodes Patients with bacteremia episodes, n Incidence rate, per 100 person-years, CI Multiple episodes 2–3 episodes ≥ 4 episodes	15 (10%) 7 (CI 4.9–10) 7 6 1	66 (17%) 11.6 (CI 9.6–13.9) 26 22 4
First episode of all-cause bacteremia Time to episode, median, days, IQR CIF at 1 year, % CIF at 2 years, % CIF at 3 years, %	410 (145–591) 4.7% (CI 1.4–8.1) 8.7% (CI 4.2–13.2) 9.7% (CI 5-14.4)	326 (118–550) 9.1% (CI 6.3–12) 14.4% (CI 10.9–17.9) 16.9% (CI 13.2–20.7)
First episode of bacteremia with OM Patients with OM, n CIF at 1 year, % CIF at 2 years, % CIF at 3 years, %	4 (3%) 1.5% (CI 0-3.4) 2.4% (CI 0-4.9) 2.6% (CI 0.1–5.1)	11 (3%) 1.7% (CI 0.4-3) 2.5% (CI 1-4.1) 2.8% (CI 1.2–4.5)

Abbreviations: *OM*, Oral microorganism; *CI*, Confidence interval; *CIF*, Cumulative incidence function

## Supplementary Information

Below is the link to the electronic supplementary material.

Supplementary Material 1

Supplementary Material 2

Supplementary Material 3

Supplementary Material 4

## Data Availability

Due to Danish data protection regulations, the authors do not have the right to share data that support findings of the study. However, statistical codes used for the analysis can be supplied upon reasonable request from the corresponding author.
