# Supplementary material for: Infective endocarditis and post-treatment bacteremia caused by oral microorganisms: a retrospective cohort study
Source: Eur J Clin Microbiol Infect Dis. 2026 May 16;45(9):2685–94. doi: 10.1007/s10096-026-05540-2 (PMC13593620; doi:10.1007/s10096-026-05540-2)
Supplement: Supplementary file 1 [file 10096_2026_5540_MOESM1_ESM.docx]

Article title: Infective endocarditis and post-treatment bacteremia caused by oral microorganisms: A retrospective cohort study

Journal name: European Journal of Clinical Microbiology & Infectious Diseases

Author names: Frederik Viktor Bang Jespersen, Simon Storgård Jensen, Merete Markvart, Henning Bundgaard, Peter L. Graversen and Emil L. Fosbøl
Affiliation and email-address: Department of Odontology, University of Copenhagen, Copenhagen, Denmark; Frederik.vb.jespersen@sund.ku.dk

**Supplementary S1: Distribution on species level according to IE index pathogens**

| **Supplementary Table 1a. Staphylococcus aureus - distribution on species level** | |
| --- | --- |
| *Staphylococcus aureus* | 215 |
| *Methicillin resistant Staphylococcus aureus (MRSA)* | 3 |

| **Supplementary Table 1b. Oral microorganism - distribution on species level** | |
| --- | --- |
| *Streptococcus mitis* | 60 |
| *Streptococcus sanguinis* | 40 |
| *Streptococcus anginosus* | 14 |
| *Streptococcus mutans* | 10 |
| *Streptococcus gordonii* | 6 |
| *Streptococcus salivarius* | 6 |
| *Streptococcus oralis* | 4 |
| *Aggregatibacter aphrophilus* | 5 |
| *Aggregatibacter actinomycetemcomitans* | 2 |
| *Abiotrophia defectiva* | 4 |
| *Haemophilus parainfluenzae* | 5 |
| *Granulicatella adiacens* | 3 |
| *Rothia dentocariosa* | 2 |
| *Cardiobacterium hominis* | 3 |
| *Granulicatella elegans* | 1 |
| *Streptococcus constellatus* | 1 |
| *Streptococcus cristatus* | 1 |
| *Gemella morbillorum* | 1 |
| *Gemella bergeri* | 1 |
| *Gemella haemolysans* | 2 |

| **Supplementary Table 1c. Other streptococcus - distribution on species level** | |
| --- | --- |
| *Streptococcus bovis* | 28 |
| *Streptococcus Group B/agalactiae* | 25 |
| *Streptococcus Group C/G* | 16 |
| *Streptococcus gallolyticus* | 8 |
| *Streptococcus pneumoniae* | 7 |
| *Streptococcus pyogenes* | 6 |

| **Supplementary Table 1d. Coagulase-negative staphylococcus (CoNS) - distribution on species level** | |
| --- | --- |
| *Staphylococcus epidermidis* | 56 |
| *Staphylococcus lugdunensis* | 6 |
| *Staphylococcus haemolyticus* | 3 |
| *Staphylococcus capitis* | 4 |
| *Staphylococcus caprae* | 1 |
| *Staphylococcus pasteuri* | 3 |
| *Staphylococcus petrasii* | 1 |
| *Staphylococcus hominis* | 1 |

| **Supplementary Table 1e. Enterococcus - distribution on species level** | |
| --- | --- |
| *Enterococcus faecalis* | 91 |
| *Enterococcus faecium* | 5 |
| *Enterococcus gallinarum* | 1 |
| *Enterococcus duran* | 1 |

| **Supplementary Table 1f. Other - distribution on species level** | |
| --- | --- |
| *Aerococcus urinae* | 10 |
| *Propionibacterium acnes* | 5 |
| *Corynebacterium striatum* | 2 |
| *Kingella kingae* | 3 |
| *Klebsiella pneumonia* | 2 |
| *Pseudomonas aeruginosa* | 2 |
| *Candida albicans* | 1 |
| *Escherichia coli* | 1 |
| *Enterobacter cloacae* | 1 |
| *Cutibacterium acnes* | 2 |
| *Lactococcus garvieae* | 2 |
| *Mycobacterium chimaera* | 2 |
| *Candida lusitaniae* | 1 |
| *Pseudomonas putida* | 1 |
| *Rickettsia* | 1 |

| **Supplementary Table 1g. Unknown pathogen - distribution** | |
| --- | --- |
| *No pathogens registered* | 55 |
| *Another* | 11 |
| *HACEK* | 1 |
