# Supplementary material for: Infective endocarditis and post-treatment bacteremia caused by oral microorganisms: a retrospective cohort study
Source: Eur J Clin Microbiol Infect Dis. 2026 May 16;45(9):2685–94. doi: 10.1007/s10096-026-05540-2 (PMC13593620; doi:10.1007/s10096-026-05540-2)
Supplement: Supplementary file 2 [file 10096_2026_5540_MOESM2_ESM.docx]

Article title: Infective endocarditis and post-treatment bacteremia caused by oral microorganisms: A retrospective cohort study

Journal name: European Journal of Clinical Microbiology & Infectious Diseases

Author names: Frederik Viktor Bang Jespersen, Simon Storgård Jensen, Merete Markvart, Henning Bundgaard, Peter L. Graversen and Emil L. Fosbøl
Affiliation and email-address: Department of Odontology, University of Copenhagen, Copenhagen, Denmark; Frederik.vb.jespersen@sund.ku.dk

**Supplementary S2: Baseline characteristics in patients eligible for bacteremia analysis stratified by index pathogen**

| Characteristics | Total | OM group | Non-OM group | Significant p-values |
| --- | --- | --- | --- | --- |
| Patients, n | 544 (100%) | 154 (28%) | 390(72%) |  |
| Men | 419 (77%) | 119 (77%) | 300 (77%) |  |
| Age, years, IQR | 66 [53, 74] | 60 [46, 71] | 68 [57, 74] | <0.001* |
| Weight, kilogram, IQR | 86 [70, 110] | 82 [67, 108] | 89 [72, 112] |  |
| Comorbidities  Chronic kidney disease  Dialysis^†^  Diabetes  COPD  Prior malignant disease  Active malignant disease  HIV/AIDS  Liver disease  Immunosuppressant drugs  Known heart failure  Native valve disease  Congenital heart disease  Previous CIED  Previous heart valve surgery  Prior IE episode | 88 (16%)  38 (7%)  111 (20%)  65 (12%)  61 (11%)  36 (7%)  4 (1%)  23 (4%)  59 (11%)  104 (19%)  60 (11%)  70 (12%)  109 (20%)  166 (31%)  32 (6%) | 10 (7%)  3 (2%)  17 (11%)  11 (7%)  11 (7%)  6 (4%)  2 (1%)  8 (5%)  9 (6%)  20 (13%)  24 (16%)  38 (25%)  19 (12%)  49 (32%)  5 (3%) | 78 (20%)  35 (9%)  94 (24%)  54 (14%)  50 (13%)  30 (8%)  2 (1%)  15 (4%)  50 (13%)  84 (22%)  36 (9%)  32 (8%)  90 (23%)  117 (30%)  27 (7%) | <0.001*  0.003*  0.05*  <0.001*  0.02* |
| Hospital stay, days, IQR | 44 [32, 53] | 38 [28, 47] | 46 [35, 56] | <0.001* |
| IE characteristics  Localization^§^  Aortic valve  Mitral valve  Tricuspid valve  Pulmonary valve  Prosthetic valve  Aortic valve  Mitral valve  Pulmonary valve  Tricuspid valve  Pacemaker cord^†^  Myocardium^†^  Modified DUKE criteria  Definite IE  Possible IE | 295 (55%)  219 (41%)  28 (5%)  29 (5%)  144 (27%)  98 (18%)  23 (4%)  26 (5%)  1 (0%)  80 (15%)  14 (2%)  646 (94%)  35 (6%) | 84 (55%)  67 (44%)  5 (3%)  17 (11%)  44 (29%)  23 (15%)  4 (3%)  17 (11%)  0 (0%)  11 (7%)  4 (3%)  150 (88%)  19 (12%) | 211 (55%)  152 (39%)  23 (6%)  12 (3%)  100 (26%)  75 (19%)  19 (5%)  9 (2%)  1 (1%)  69 (18%)  10 (2%)  496 (96%)  16 (4%) | 0.001*  <0.001*  <0.001*  <0.001* |
| Suspected focus of infection  Any suspected focus  Dental focus  Spondylodiscitis  Other osseus focus  Thorax  Abdomen  Urinary tract  IV catheter  Dialysis catheter  Skin  Other primary focus | 351 (65%)  139 (26%)  50 (9%)  25 (5%)  89 (17%)  30 (6%)  65 (12%)  29 (5%)  16 (3%)  55 (10%)  39 (7%) | 83 (54%)  51(35%)  9 (6%)  6 (4%)  23 (15%)  5 (3%)  6 (4%)  5 (3%)  3 (2%)  7 (5%)  3 (2%) | 268 (69%)  88(23%)  41(11%)  19 (5%)  66 (17%)  25 (6%)  59 (15%)  24 (6%)  13 (3%)  48 (12%)  36 (9%) | 0.006*  0.03*  0.03*  0.03*  0.01* |
| Embolism at admission  Central nervus system  Liver  Spleen  Lungs  Peripheral vasculature  Eye  Other | 75 (14%)  51 (10%)  1 (0%)  13 (2%)  4 (1%)  10 (2%)  2 (0%)  7 (1%) | 17 (11%)  12 (8%)  1 (1%)  2 (1%)  2 (1%)  2 (1%)  0 (0%)  1 (1%) | 58 (15%)  39 (10%)  0 (0%)  11 (3%)  2 (1%)  8 (2%)  2 (1%)  6 (2%) |  |
| Complications during hospitalization  Embolism  Heart failure  Conduction abnormalities  Persistence of bacteremia  Worsening valvular insufficiency  Sepsis  Dialysis  Admission to ICU  Heart-surgery | 89 (16%)  42 (8%)  79 (15%)  27 (5%)  79 (15%)  71 (13%)  47 (9%)  132 (24%)  342 (63%) | 19 (12%)  16 (10%)  29 (19%)  7 (5%)  35 (23%)  13 (8%)  8 (5%)  36 (23%)  106 (69%) | 70 (18%)  26 (7%)  50 (13%)  20 (5%)  44 (11%)  58 (15%)  39 (10%)  96 (25%)  236 (61%) | 0.003* |
| Functional status during hospitalization  Walking aid necessary  Patient-lift necessary  Enteral/parenteral nutrition | 283 (52%)  119 (22%)  143 (26%) | 58 (38%)  23 (15%)  28 (18%) | 225 (58%)  96 (25%)  115 (30%) | <0.001*  0.05* |
| Behavioral factors  Active smoking  Active drinking  Active IV abuse | 122 (22%)  374 (69%)  11 (2%) | 30 (20%)  103 (67%)  3 (2%) | 92 (24%)  271 (70%)  8 (2%) |  |
| In hospital oral examination | 370 (68%) | 120 (78%) | 250 (64%) | <0.001* |

Abbreviations: OM, Oral microorganisms; ESC, European Society of Cardiology; IE, Infective endocarditis; COPD, Chronic obstructive pulmonary disease; CIED, Cardiac Implantable Electronic Device; ICU, Intensive Care Unit.

^*^Test results of Chi-square, Fisher’s exact, or Mann-Whitney U tests when appropriate. Significance is reported at p < 0.05 with Benjamini–Hochberg adjusted p-values.

^†^Variable with more than 5% missing value.

^§^Numbers exceed total number of patients, as some patients had involvement of multiple valves
