# Supplementary material for: Infective endocarditis and post-treatment bacteremia caused by oral microorganisms: a retrospective cohort study
Source: Eur J Clin Microbiol Infect Dis. 2026 May 16;45(9):2685–94. doi: 10.1007/s10096-026-05540-2 (PMC13593620; doi:10.1007/s10096-026-05540-2)
Supplement: Supplementary file 4 [file 10096_2026_5540_MOESM4_ESM.docx]

Article title: Infective endocarditis and post-treatment bacteremia caused by oral microorganisms: A retrospective cohort study

Journal name: European Journal of Clinical Microbiology & Infectious Diseases

Author names: Frederik Viktor Bang Jespersen, Simon Storgård Jensen, Merete Markvart, Henning Bundgaard, Peter L. Graversen and Emil L. Fosbøl
Affiliation and email-address: Department of Odontology, University of Copenhagen, Copenhagen, Denmark; [Frederik.vb.jespersen@sund.ku.dk](mailto:Frederik.vb.jespersen@sund.ku.dk)

**Supplementary S4: Microorganisms identified in first episode of all-cause bacteremia and bacteremia with oral microorganisms**

| Distribution of pathogens | First episode of all-cause bacteremia | | First episode of bacteremia with OM | |
| --- | --- | --- | --- | --- |
|  | All pathogens^*^ | Pathogens same as index IE | All pathogens^§^ | Pathogens same as index IE |
| Total, patients, n | 81 | 25 | 15 | 2 |
| *Staphylococcus aureus*, n (%) | 15 (19%) | 11 (44%) | 1 (7%)^†^ | 1 (50%)^†^ |
| OM, n (%)   - *Streptococcus mitis* - *Streptococcus sanguis* - *Abiotrophia defectiva* - *Fusobacterium nucleatum* - *Peptostreptococcus spp.* - *Streptococcus mutans* - *Streptococcus salivarius* | 7 (9%) 4 (5%) 1 (1%) 1 (1%) 1 (1%) 1 (1%) 1 (1%) | 1 (4%) 1 (4%) | 7 (47%) 4 (27%) 1 (7%) 1 (7%) 1 (7%) 1 (7%) 1 (7%) | 1 (50%) 1 (50%) |
| Other streptococci, n (%)   - *Streptococcus bovis* - *Streptococcus agalactiae gr. b* - *Streptococcus pyogenes* - *Streptococcus dysgalactiae* | 3 (4%) 2 (2%) 2 (2%) 1 (1%) |  |  |  |
| CoNS, n (%)   - *Staphylococcus epidermidis* - *Staphylococcus capitis* - *Coagulase-negative staphylococcus (unspecified)* | 10 (12%) 1 (1%) 1 (1%) | 4 (16%) |  |  |
| Enterococcus, n (%)   - *Enterococcus faecalis* - *Enterococcus faecium* - *Enterococcus gallinarum* | 11 (14%) 2 (2%) 1 (1%) | 6 (24%)  1 (4%) |  |  |
| Other, n (%)   - *Escherichia coli* - *Enterobacter cloacae* - *Aerococcus urinae* - *Klebsiella pneumonia* - *Citrobacter koseri* - *Klebsiella oxytoca* - *Morganella morganii* - *Proteus mirabilis* - *Rothia species* - *Pseudomonas aeruginosa* | 7 (9%) 4 (5%) 2 (2%) 2 (2%) 1 (1%) 1 (1%) 1 (1%) 1 (1%) 1 (1%) 1 (1%) | 1 (4%) |  |  |

Abbreviations: OM, Oral microorganisms; IE, Infective endocarditis; CoNS, Coagulase-negative *Staphylococcus*
^*^Numbers exceed 81 bacteremia cases as some patients had multiple pathogens isolated.
^§^Numbers exceed 15 bacteremia cases as one patient had *Fusobacterium nucleatum* and *Peptostreptococcus spp.* isolated and one patient had *Staphylococcus aureus* and *Streptococcus mitis* isolated
^†^One patient had *Streptococcus mitis* and *Staphylococcus aureus* isolated
